# High-Sensitivity Cardiac Troponin Concentration and Risk of First-Ever Cardiovascular Outcomes in 154,052 Participants

**DOI:** 10.1016/j.jacc.2017.05.062

**Published:** 2017-08-01

**Authors:** Peter Willeit, Paul Welsh, Jonathan D.W. Evans, Lena Tschiderer, Charles Boachie, J. Wouter Jukema, Ian Ford, Stella Trompet, David J. Stott, Patricia M. Kearney, Simon P. Mooijaart, Stefan Kiechl, Emanuele Di Angelantonio, Naveed Sattar

**Affiliations:** a Department of Neurology, Medical University of Innsbruck, Innsbruck, Austria; b Department of Public Health and Primary Care, University of Cambridge, Cambridge, United Kingdom; c Institute of Cardiovascular and Medical Sciences, University of Glasgow, Glasgow, United Kingdom; d Transplant Unit, Papworth Hospital NHS Foundation Trust, Papworth Everard, Cambridge, United Kingdom; e Department of Cardiology, Leiden University Medical Center, Leiden, the Netherlands; f Robertson Centre for Biostatistics, University of Glasgow, Glasgow, United Kingdom; g Department of Gerontology and Geriatrics, Leiden University Medical Center, Leiden, the Netherlands; h Department of Epidemiology and Public Health, University College Cork, Cork, Ireland; i National Institute for Health Research Blood and Transplant Research Unit in Donor Health and Genomics, University of Cambridge, Cambridge, United Kingdom; j NHS Blood and Transplant, Cambridge, United Kingdom; k British Heart Foundation Cambridge Centre of Excellence, University of Cambridge, Cambridge, United Kingdom

**Keywords:** biomarker, cardiovascular disease, coronary heart disease, primary prevention, stroke, systematic review, CHD, coronary heart disease, CI, confidence interval, CRP, C-reactive protein, CVD, cardiovascular disease, eGFR, estimated glomerular filtration rate, HDL-C, high-density lipoprotein cholesterol, HR, hazard ratio, hs-cTnI, high-sensitivity cardiac troponin I, hs-cTnT, high-sensitivity cardiac troponin T, MI, myocardial infarction, NRI, net reclassification index, NT-proBNP, N-terminal pro–B-type natriuretic peptide, RR, relative risk

## Abstract

**Background:**

High-sensitivity assays can quantify cardiac troponins I and T (hs-cTnI, hs-cTnT) in individuals with no clinically manifest myocardial injury.

**Objectives:**

The goal of this study was to assess associations of cardiac troponin concentration with cardiovascular disease (CVD) outcomes in primary prevention studies.

**Methods:**

A search was conducted of PubMed, Web of Science, and EMBASE for prospective studies published up to September 2016, reporting on associations of cardiac troponin concentration with first-ever CVD outcomes (i.e., coronary heart disease [CHD], stroke, or the combination of both). Study-specific estimates, adjusted for conventional risk factors, were extracted by 2 independent reviewers, supplemented with de novo data from PROSPER (Pravastatin in Elderly Individuals at Risk of Vascular Disease Study), then pooled by using random effects meta-analysis.

**Results:**

A total of 28 relevant studies were identified involving 154,052 participants. Cardiac troponin was detectable in 80.0% (hs-cTnI: 82.6%; hs-cTnT: 69.7%). In PROSPER, positive associations of log-linear shape were observed between hs-cTnT and CVD outcomes. In the meta-analysis, the relative risks comparing the top versus the bottom troponin third were 1.43 (95% confidence interval [CI]: 1.31 to 1.56) for CVD (11,763 events), 1.67 (95% CI: 1.50 to 1.86) for fatal CVD (7,775 events), 1.59 (95% CI: 1.38 to 1.83) for CHD (7,061 events), and 1.35 (95% CI: 1.23 to 1.48) for stroke (2,526 events). For fatal CVD, associations were stronger in North American studies (p = 0.010) and those measuring hs-cTnT rather than hs-cTnI (p = 0.027).

**Conclusions:**

In the general population, high cardiac troponin concentration within the normal range is associated with increased CVD risk. This association is independent of conventional risk factors, strongest for fatal CVD, and applies to both CHD and stroke.

Cardiac troponins are structural proteins in the contractile apparatus of cardiac myocytes that are released into the circulation after cardiac myocyte cell death (1). Since the introduction of the first troponin assays in the early 1990s, measurement of circulating cardiac troponin concentration has become a cornerstone in the diagnosis and acute management of myocardial infarction (MI) (2). Development of high-sensitivity assays allowed the detection of cardiac troponin I and T (hs-cTnI and hs-cTnT, respectively) at lower concentrations, which enabled earlier identification of myocardial injury and increased diagnostic accuracy in patients with suspected MI (3).

At the same time, these methodological advances have led to a fundamental change in the clinical interpretation of cardiac troponin assay results. Although patients traditionally have been classified as being “troponin-positive” or “troponin-negative” in the context of the diagnosis of acute MI, new-generation assays allow a continuous interpretation of findings (i.e., higher vs. lower concentration). Furthermore, high-sensitivity assays can detect circulating cardiac troponin in individuals with no clinically manifest myocardial damage or previous cardiovascular disease (CVD).

Recent studies in subjects recruited from the general population have suggested that elevations in circulating cardiac troponin are associated with a higher risk of a first-ever CVD event 4, 5, 6, 7, 8, 9, 10, 11, 12, 13, 14, 15, 16, 17, 18, 19, 20, 21, 22, 23, 24, 25, 26, 27, 28, 29, 30. The largest such study, from the BiomarCaRE (Biomarkers for Cardiovascular Risk Assessment in Europe) consortium, reported a hazard ratio (HR) for CVD of 1.92 (95% confidence interval [CI]: 1.76 to 2.10) comparing the highest versus the lowest quintile of hs-cTnI concentration (11). Although these studies have yielded promising findings, their interpretation and comparison have been hampered by use of different scales and inconsistent adjustment of effect estimates. To clarify associations of cardiac troponins with incident CVD, the present study collated effect estimates from published studies conducted in populations free of previous CVD, supplemented them with de novo data, and combined them in a meta-analysis.

## Methods

We systematically searched the electronic databases PubMed, Web of Science, and EMBASE for prospective studies published up to September 2016 that reported on associations of cardiac troponin concentration with incident CVD outcomes. The search strategy is detailed in Online Table 1. Studies were eligible for inclusion if they: 1) had measured hs-cTnI and/or hs-cTnT with a high-sensitivity assay; 2) had recruited participants not based on having a history of CVD at baseline; and 3) had recorded CVD outcomes over more than 1 year of follow-up. We further obtained unpublished tabular data from the Bruneck Study through direct correspondence with study investigators (31). Using standardized data extraction protocols, 2 independent reviewers (P.W. and J.D.W.E.) extracted, by consensus, information on geographical location, baseline survey dates, study design, exclusion of participants with pre-existing CVD, mean age at baseline, proportion of male subjects, proportion of white participants, sample type, cardiac troponin assay manufacturer, assay detection limit, proportion of participants with detectable cardiac troponin, and number of participants in the study. In relation to incident outcomes, the reviewers extracted data on duration of follow-up, the specific composition of reported endpoints, the number of outcomes accrued, effect sizes, and the degree of statistical adjustment of any reported association. The degree of adjustment was classified as “o” when relative risks (RRs) were adjusted for age and sex only, “+” when RRs were adjusted for conventional risk factors (age, sex, smoking, diabetes, blood pressure, and total and high-density lipoprotein cholesterol [HDL-C]); “++” when RRs were also adjusted for C-reactive protein (CRP) or serum creatinine; and “+++” after further adjustment for B-type natriuretic peptides. If multiple publications on the same study were available, the most up-to-date or comprehensive information was used. Methods and results are reported in accordance with the Preferred Reporting Items for Systematic Review and Meta-Analysis Protocols guidelines (32).

### The PROSPER study

PROSPER (Pravastatin in Elderly Individuals at Risk of Vascular Disease Study) was a randomized, double-blind, placebo-controlled pravastatin trial in individuals with pre-existing CVD or risk factors thereof (i.e., smoking, hypertension, diabetes) 33, 34. The present analysis involved 4,402 participants with no previous CVD and with complete information on hs-cTnT and covariates, recruited at study centers in Scotland, Ireland, and the Netherlands (Online Figure 1). Measurement of hs-cTnT used plasma samples obtained 6 months after randomization using a high-sensitivity electrochemiluminescence immunoassay. The occurrence of incident outcomes was adjudicated by the PROSPER Endpoints Committee during the in-trial phase (mean duration: 3.2 years) and ascertained with routine health data thereafter. The institutional ethics review boards of all centers approved the protocol, and all participants gave written informed consent. The protocol adhered to the principles of the Declaration of Helsinki. Further details are provided in the Online Appendix.

### Statistical analysis

The statistical analysis was conducted according to a pre-defined statistical analysis plan. The combined CVD endpoint was defined as fatal coronary heart disease (CHD), nonfatal MI, and fatal plus nonfatal stroke (ischemic, hemorrhagic, or unclassified). In PROSPER, we assessed cross-sectional associations between hs-cTnT and other characteristics by using linear and logistic regression models (for continuous and categorical data, respectively), adjusted for age, sex, center, and treatment arm. HRs were calculated for CVD outcomes stratified according to treatment arm and were progressively adjusted for age, sex, and center (model 1), plus smoking, history of diabetes mellitus, history of hypertension, systolic blood pressure, total cholesterol, HDL-C, and body mass index (model 2), plus CRP, estimated glomerular filtration rate (eGFR), and N-terminal pro–B-type natriuretic peptide (NT-proBNP) (model 3).

To characterize shapes of associations with CVD outcomes, HRs were calculated across quartiles of detectable hs-cTnT concentration by using participants with undetectable hs-cTnT as the reference group, and HRs were plotted against the median hs-cTnT concentration within each category. For the outcomes of CVD and fatal CVD, effect modification was explored across clinically relevant subgroups with formal tests of interaction. We also tested whether assessment of hs-cTnT, in addition to age, sex, smoking status, history of diabetes mellitus, systolic blood pressure, and levels of total cholesterol and HDL-C, could improve the prediction of 10-year CVD risk. C-index changes as well as categorical and continuous net reclassification indexes (NRIs) were calculated by using previously published methods 35, 36. Categorical NRIs were calculated across 10-year predicted risk categories of <15%, 15% to <25%, and ≥25%, chosen to reflect the elevated risk in the study population; 95% CIs were estimated for these prediction metrics by using bootstrap resampling with 999 repetitions.

Study-specific risk estimates were pooled by using random effects meta-analyses. Odds ratios and HRs were assumed to approximate the same underlying measure of RR. When studies reported RRs of various levels of adjustment, the most adjusted estimate was used. To enable a consistent approach to analysis, RRs and 95% CIs in each study were standardized to a common scale (i.e., to reflect a comparison of the top third vs. the bottom third of the population's cardiac troponin distribution, using methods previously described) (37). Consistency of findings across studies was assessed with standard chi-square tests and the *I*^2^ statistic (38). Subgroup analyses were conducted by using meta-regression across pre-specified study-level characteristics (39). Evidence of publication bias was assessed by using funnel plots and Egger's asymmetry test (40). Statistical tests were 2-sided and used a significance level of p < 0.05 for principal analyses and p < 0.01 for subgroup analyses.

## Results

In PROSPER, hs-cTnT was detectable in 3,853 (87.5%) of 4,402 participants. The median concentration of hs-cTnT was 7 ng/l (interquartile range: 4 to 11 ng/l), and 85% of hs-cTnT values were within the normal range of ≤14 ng/l. Concentration of hs-cTnT correlated positively with age, male sex, body mass index, blood pressure, NT-proBNP, CRP, history of diabetes mellitus, and history of hypertension (Online Table 2). Concentration of hs-cTnT was lower in current smokers and correlated inversely with renal function assessed by eGFR. There was no difference in hs-cTnT according to trial arm.

**Figure 1:**
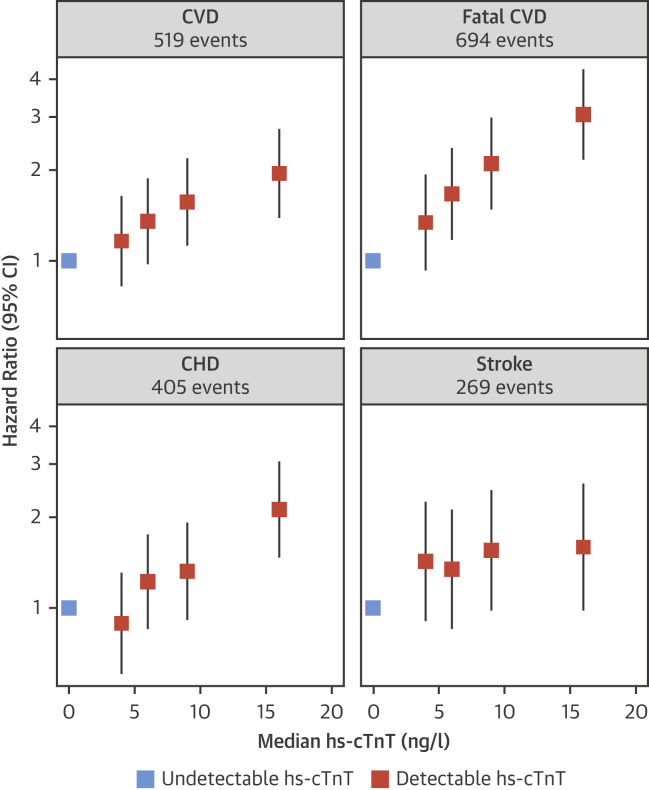
PROSPER: hs-cTnT Associations With CVD Outcomes After multivariable adjustment, models were stratified according to treatment arm; the group with undetectable high-sensitivity cardiac troponin T (hs-cTnT) values was used as the reference group. The median hs-cTnT concentrations (ranges) in quartiles of detectable hs-cTnT were 4 ng/l (3 to 4), 6 ng/l (5 to 7), 9 ng/l (8 to 11), and 16 ng/l (12 to 1,840). **Sizes of data markers** are proportional to the inverse of the variance of the hazard ratios. CHD = coronary heart disease; CI = confidence interval; CVD = cardiovascular disease. PROSPER = Pravastatin in Elderly Individuals at Risk of Vascular Disease Study.

In the overall cohort, 694 fatal CVD events were recorded over a follow-up duration of up to 11.3 years (mean: 8.2 years). At the Scottish center, 519 fatal and nonfatal CVD events, 405 CHD events, and 269 stroke events were recorded. After adjusting for age, sex, smoking, history of diabetes mellitus, history of hypertension, systolic blood pressure, total cholesterol, HDL-C, and body mass index, the shapes of associations between baseline hs-cTnT concentration and CVD outcomes were approximately log linear (Figure 1). The adjusted HRs for a comparison of the top third versus the bottom third of the hs-cTnT concentration were 1.55 (95% CI: 1.23 to 1.96) for CVD, 2.16 (95% CI: 1.74 to 2.67) for fatal CVD, 1.85 (95% CI: 1.42 to 2.42) for CHD, and 1.21 (95% CI: 0.88 to 1.67) for stroke. There was some attenuation of estimates after further adjustment for CRP, eGFR, and NT-proBNP (Online Table 3). There was no evidence for effect modification by a range of participant characteristics, including age, sex, diabetes, eGFR, and NT-proBNP, but there was evidence for a somewhat stronger association in never- or ex-smokers compared with current smokers (Online Figure 2).

### Literature-based meta-analysis

**Figure 2:**
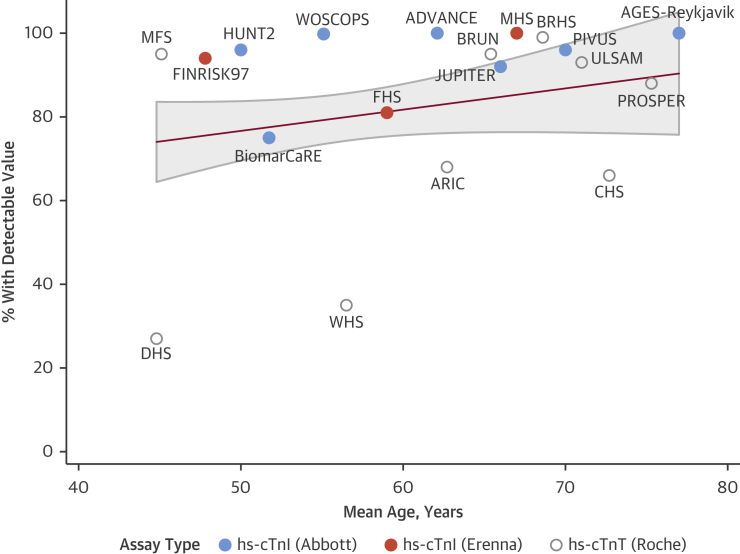
Detectable Cardiac Troponin Concentrations The percentage of participants with detectable cardiac troponin concentrations was categorized by mean age and assay type per included study. The **red line** and **gray area** represent the line of best fit and 95% confidence interval, respectively, with individual studies being weighted according to their number of participants. ADVANCE = Atherosclerotic Disease, Vascular Function and Genetic Epidemiology Study; AGES-Reykjavik = Age, Gene/Environment Susceptibility-Reykjavik Study; ARIC = Atherosclerosis Risk in Communities Study; BiomarCaRE = Biomarkers for Cardiovascular Risk Assessment in Europe; BRHS = British Regional Heart Study; BRUN = Bruneck Study; CHS = Cardiovascular Health Study; DHS = Dallas Heart Study; FHS = Framingham Heart Study; FINRISK97 = FINRISK 1997 Survey; hs-cTnT = high-sensitivity cardiac troponin T; HUNT2 = Nord-Trondelag Health Study 2; JUPITER = Justification for the Use of Statins in Prevention: An Intervention Trial Evaluating Rosuvastatin Trial; MFS = MIDSPAN Family Study; MHS = Minnesota Heart Study; PIVUS = Prospective Investigation of the Vaculature in Uppsala Seniors Study; PROSPER = Pravastatin in Elderly Individuals at Risk of Vascular Disease Study; ULSAM = Uppsala Longitudinal Study of Adult Men; WHS = Women’s Health Study; WOSCOPS = West of Scotland Coronary Prevention Study.

We screened 4,645 records and identified 28 eligible prospective studies 4, 5, 6, 7, 8, 9, 10, 11, 12, 13, 14, 15, 16, 17, 18, 19, 20, 21, 22, 23, 24, 25, 26, 27, 28, 29, 30 reporting on a total of 154,052 participants (Table 1, Online Figure 3). Eighteen studies were based in Europe, 7 in North America, 1 in Asia, and 2 were multinational. The mean age was 56.1 years, 52.8% of participants were male, and 88.6% were white. Seventeen studies had measured hs-cTnI and 11 had measured hs-cTnT. The proportion of participants with detectable cardiac troponin concentrations was 80.0% overall (82.6% in hs-cTnI studies and 69.7% in hs-cTnT studies). Multivariable regression analyses weighting studies according to their size identified that the proportion of participants with detectable troponin was higher in hs-cTnI studies (p = 0.007) and in older study populations (8.3% per decade higher mean age; p = 0.009) but was unrelated to sex distributions (p = 0.155). Figure 2 displays the proportion of troponin detection according to study, assay type, and mean age of the study population.

**Table 1:** Characteristics of Prospective Studies

Study (Ref. #)	Location	Baseline Survey Date (Range)	Study Design	Mean Follow-Up, Yrs	Mean Age, Yrs	Male, %	White, %	Type of Troponin (Assay)	Sample Type	Detection Limit, ng/l	Detectable Troponin, %	Participants, n	No. of Events			
													CVD	CHD	Stroke	Fatal CVD
ADVANCE (4)	United States	2001–2004	PC	11.3∗	62.1	50.8	62.1	hs-cTnI (Abbott)	Serum	1.2	100	1,135	–	164	–	–
AGES-Reykjavik (5)	Iceland	2002–2006	PC	8.2∗	77.0	42.5	100.0	hs-cTnI (Abbott)	Serum	1.2	100	5,691	957	716	–	–
ARIC 6, 7, 8, 9	United States	1996–1998	PC	12.1	62.7	43.8	78.3	hs-cTnT (Roche)	Plasma	3.0	68	10,350	610	527	507	358
BRHS (10)	United Kingdom	1998–2000	PC	13.0∗	68.6	100.0	99.0	hs-cTnT (Roche)	Plasma	3.0	99	2,715	475	–	–	–
BRIANZA (11)	Italy	1986–1993	PC	15.0∗	46.7∗	49.3	NR	hs-cTnI (Abbott)	Serum	1.9	75	4,932	393	–	–	167
BRUN	Italy	2000	PC	10.0†	65.4	46.1	100.0	hs-cTnT (Roche)	Serum	12.0	95	642	74	33	37	49
CAERPHILLY (11)	United Kingdom	1989–1993	PC	22.2†	62.4∗	100.0	100.0	hs-cTnI (Abbott)	Serum	1.9	75	2,171	583	–	–	470
CHS 12, 13	United States	1989–1993	PC	11.8∗	72.7	40.5	83.8	hs-cTnT (Roche)	Serum	3.0	66	4,221	–	–	NR	1,103
CIRCS 12, 13	Japan	2001–2011	NCC	2.0∗	38.0–86.0‡	68.0	0.0	hs-cTnT (Roche)	Serum	3.0	NR	360	–	120	–	–
DAN-MONICA 12, 13	Denmark	1982–1992	PC	29.0†	50.0∗	50.6	NR	hs-cTnI (Abbott)	Serum	1.9	75	7,582	1,326	-	–	1,002
DHS 12, 13	United States	2000–2002	PC	6.4∗	44.8	44.1	29.4	hs-cTnT (Roche)	Serum	3.0	27	3,459	–	–	–	59
FHS 12, 13	United States	1995–1998	PC	11.3	59.0	46.9	100.0	hs-cTnI (Erenna)	Plasma	0.2	81	3,265	334	173	–	–
FINRISK97 12, 13	Finland	1997	PC	14.0†	47.8∗	49.7	100.0	hs-cTnI (Erenna)	Serum	1.0	94	7,899	770	363	299	–
HUNT2 18, 19	Norway	1995–1997	PC	13.9	50.0∗	45.6	97.0	hs-cTnI (Abbott)	Serum	1.2	96	9,712	–	292	–	708
JUPITER 18, 19	Multinational	2003–2006	PC§	2.0∗	66.0∗	67.7	81.8	hs-cTnI (Abbott)	Plasma	1.9	92	12,956	304	224	70	46
KORA 11, 21	Germany	1994–2001	PC/CCoh∥	14.5†	50.5∗	49.7	NR	hs-cTnI (Abbott)	Serum	1.9	75	8,913	525	803	–	331
MFS 11, 21	United Kingdom	1996	PC	17.3∗	45.1	44.5	100.0	hs-cTnT (Roche)	Plasma	3.0	95	1,721	135	–	–	–
MHS 11, 21	United States	1990–1997	NCC	15.0†	67.0	62.1	97.0	hs-cTnI (Erenna)	Serum	NR	100	464	–	–	–	211
MOLI-SANI 11, 21	Italy	2005–2010	PC	4.2∗	54.6∗	48.1	NR	hs-cTnI (Abbott)	Serum	1.9	75	24,325	473	–	–	151
PIVUS 23, 24	Sweden	2001	PC	10.0∗	70.0	50.0	100.0	hs-cTnI (Abbott)	Plasma	1.5	96	1,004	163	–	–	37
PREVEND 23, 24	Netherlands	1997	PC	12.0†	49.3	49.8	94.9	hs-cTnT (Roche)	Plasma	3.0	NR	8,121	–	583	–	–
PRIME-BEL 23, 24	United Kingdom	1990–1993	PC	12.0†	54.7∗	100.0	NR	hs-cTnI (Abbott)	Serum	1.9	75	2,745	505	–	–	149
PROSPER	Multinational	1997–1999	PC§	8.2	75.3	44.8	100.0	hs-cTnT (Roche)	Plasma	3.0	88	4,402	519	405	269	694
SHHEC 11, 29	United Kingdom	1984–1995	PC	20.0	48.9	50.4	NR	hs-cTnI (Abbott)	Serum	1.9	75	16,000	2,953	1,980	797	1,786
SHIP 11, 29	Germany	1997–2001	PC	11.0†	50.0∗	48.7	NR	hs-cTnI (Abbott)	Serum	1.9	75	3,871	–	–	–	38
ULSAM 11, 29	Sweden	1991–1995	PC	10.0∗	71.0	100.0	100.0	hs-cTnT (Roche)	Plasma	3.0	93	561	148	86	62	46
WHS 11, 29	United States	1992–1995	PC/CCoh§¶	12.3∗	56.5∗	0.0	90.8	hs-cTnT (Roche)	Plasma	3.0	35	1,517	516	176	272	119
WOSCOPS 11, 29	United Kingdom	1989–1991	PC§	15.0†	55.1	100.0	NR	hs-cTnI (Abbott)	Plasma	1.2	99.8	3,318	–	413	213	251
Total		1982–2011		11.9	56.1	52.8	88.6				80.0	154,052	11,763	7,061	2,526	7,775

Values are ranges, weighted means, or sums, unless otherwise indicated.

ADVANCE = Atherosclerotic Disease, Vascular Function and Genetic Epidemiology Study; AGES-Reykjavik = Age, Gene/Environment Susceptibility-Reykjavik Study; ARIC = Atherosclerosis Risk in Communities Study; BRHS = British Regional Heart Study; BRIANZA = MONICA Brianza Study; BRUN = Bruneck Study; CAERPHILLY = Caerphilly Prospective Study; CCoh = case-cohort study; CHD = coronary heart disease; CHS = Cardiovascular Health Study; CIRCS = Circulatory Risk in Communities Study; CVD = cardiovascular disease; DAN-MONICA = Danish MONICA Study; DHS = Dallas Heart Study; FHS = Framingham Heart Study; FINRISK97 = FINRISK 1997 Survey; hs-cTnI = high-sensitivity cardiac troponin I; hs-cTnT = high-sensitivity cardiac troponin T; HUNT2 = Nord-Trondelag Health Study 2; JUPITER = Justification for the Use of Statins in Prevention: An Intervention Trial Evaluating Rosuvastatin Trial; KORA = Kooperative Gesundheitsforschung in der Region Augsburg; MFS = MIDSPAN Family Study; MHS = Minnesota Heart Study; MOLI-SANI = Moli-Sani Project; NCC = nested case-control study; NR = not reported; PC = prospective cohort study; PIVUS = Prospective Investigation of the Vasculature in Uppsala Seniors Study; PREVEND = Prevention of Renal and Vascular End Stage Disease Study; PRIME-BEL = Prospective Epidemiological Study of Myocardial Infarction from Belfast; PROSPER = Pravastatin in Elderly Individuals at Risk of Vascular Disease Study; SHHEC = Scottish Heart Health Study and Scottish MONICA; SHIP = Study of Health in Pomerania; ULSAM = Uppsala Longitudinal Study of Adult Men; WHS = Women’s Health Study; WOSCOPS = West of Scotland Coronary Prevention Study.

∗ Median.

† Maximum.

‡ Range.

§ Prospective study was nested in a trial.

∥ The KORA study reported on the association with CHD events in a case-cohort dataset (2,748 participants, maximum follow-up of 16 years, hs-cTnI measured with the Erenna assay).

¶ The WHS investigated results separately for participants with and without diabetes (using a prospective study and case-cohort design, respectively).

**Figure 3:**
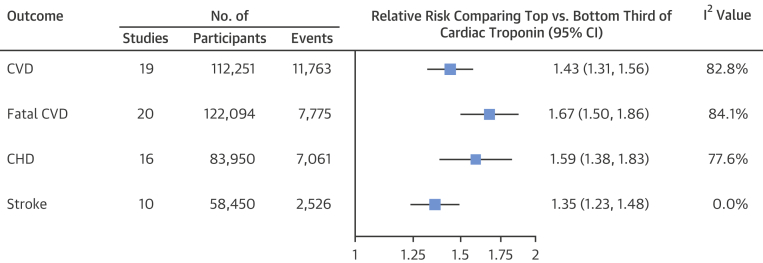
Combined Adjusted Relative Risk for CVD Outcomes Study-specific relative risks were pooled by using random effects meta-analysis and compared in the top third versus the bottom third of cardiac troponin concentration. Abbreviations as in Figure 1.

The combined RR in the top third versus the bottom third of cardiac troponin concentration was 1.43 for CVD, 1.67 for fatal CVD, 1.59 for CHD, and 1.35 for stroke (Figure 3). Forest plots for each outcome are shown in Online Figures 4 to 7. The level of between-study heterogeneity was low for stroke (*I*^2^ = 0%; p = 0.764) and high for other outcomes (*I*^2^ >75%; all p < 0.001). Magnitudes of associations with CVD and fatal CVD were largely similar across studies grouped according to geographical regions, in studies nested versus those not nested in clinical trials (Online Figure 8), and for different mean ages of study participants, proportions of male participants, and numbers of recorded events (Online Figure 9). Associations for fatal CVD were somewhat stronger in studies based in North America (p = 0.010) (Online Figure 8) and studies measuring hs-cTnT rather than hs-cTnI (p = 0.027) (Online Figure 8). Associations with CVD were stronger in studies with a higher proportion of nonwhite participants (p = 0.004) (Online Figure 9). There was evidence for publication bias in reporting associations with fatal CVD (p_Egger_ = 0.027) but not for the other outcomes assessed (Online Figure 10).

The addition of hs-cTnT concentration to a prediction model containing information on conventional risk factors improved the prediction of fatal CVD (Online Table 4). It improved the C-index by 0.028 (95% CI: 0.007 to 0.050; p = 0.018), the categorical NRI by 0.123 (95% CI: 0.074 to 0.153; p < 0.001), and the continuous NRI by 0.357 (95% CI: 0.277 to 0.436; p < 0.001). In contrast, for the overall CVD outcome, smaller improvements in the continuous NRI of 0.152 (95% CI: 0.052 to 0.253; p = 0.003) were observed, whereas no improvements were observed in the C-index (p = 0.51) and the categorical NRI (p = 0.25). Detailed reclassification data are provided in Online Table 5.

## Discussion

In the present report that combined de novo data from the PROSPER study with a comprehensive literature-based meta-analysis, we reliably quantified associations of baseline cardiac troponin measurements with first-ever CVD outcomes (Central Illustration). We further determined the added predictive value of such measurements in the primary prevention of CVD.

**Central Illustration:**
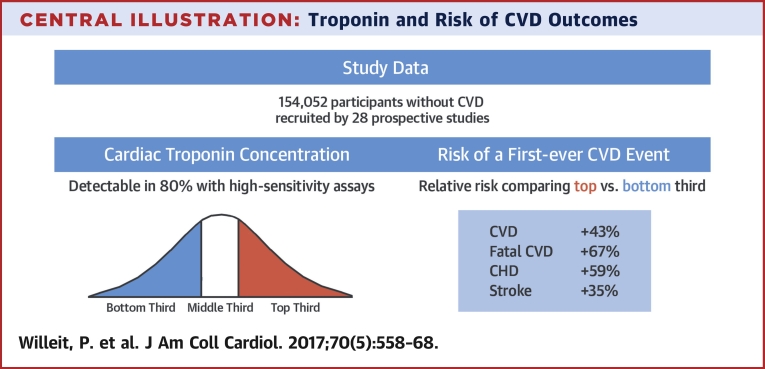
Troponin and Risk of CVD Outcomes In assessing the association of cardiac troponin concentration and CVD outcomes, we identified 28 relevant studies. Thirds of cardiac troponin concentration were defined within each study separately. Study-specific relative risks for first-ever CVD outcomes were pooled by using a random effects meta-analysis, demonstrating that high cardiac troponin levels in the normal range are associated with increased CVD event risk. The distribution graph is illustrative and cardiac troponin distributions were not normally distributed. CHD = coronary heart disease; CVD = cardiovascular disease.

### Associations with CVD outcomes

Overall, the present systematic review and meta-analysis involved data from 28 long-term prospective studies with a total of 154,052 participants without previous CVD. All included studies measured cardiac troponins hs-cTnT or hs-cTnI with high-sensitivity assays and reported detectable levels in 80% of people, on average. Pooled estimates from the meta-analysis suggest that individuals with cardiac troponin values in the top third of the population distribution are at 43% increased risk of any CVD, 59% increased risk of CHD, and 67% increased risk of fatal CVD outcomes. A smaller but significant increase in stroke risk (35%) was noted. It is likely that this association was driven by cardiac and heart rhythm abnormalities causing ischemic stroke, including paroxysmal atrial fibrillation (41). An analysis of the Atherosclerosis Risk in Communities Study according to stroke subtype revealed preferential associations of hs-cTnT with cardioembolic stroke (8). Furthermore, Wrigley et al. (42) recently reported that, in patients with acute ischemic stroke, an elevated troponin concentration was associated with structural cardiac disease detected by using echocardiography.

Our findings in the meta-analysis were further substantiated by analyses of PROSPER study data, showing that subjects with higher blood concentrations of hs-cTnT were at higher risk of developing fatal and nonfatal CVD and CHD. Associations were independent of conventional CVD risk factors and persisted even after additional adjustment for NT-proBNP, eGFR, and CRP. We could also confirm a previous suggestion from the BiomarCaRE consortium (11) that the risk of these diseases increased approximately log linearly with higher cardiac troponin concentration, with no evidence for a threshold effect at a certain level. It is striking that we observed significant positive associations with CVD outcomes even though 85% of the study population had hs-cTnT values within the normal range (≤14 ng/l) at baseline.

We also detected significant between-study heterogeneity for the associations with the outcomes of all CVD and fatal CVD. Studies varied in their age and sex profile, outcome definitions, follow-up durations, and degrees of statistical adjustment, but none of these factors had an influence on the strengths of associations observed. However, our analysis revealed possibly stronger associations in studies based in North America and with a higher proportion of nonwhite participants. Although it is well established that nonwhite participants have higher cardiac troponin values compared with white participants 4, 7, 8, 12, 15, single studies have so far not detected that associations of cardiac troponins with CHD (7) or stroke (8) are modified according to ethnicity. Furthermore, we noted a trend toward stronger associations for hs-cTnT than for hs-cTnI. To shed more light on this preliminary finding, future research is needed that evaluates, in a direct comparison, whether the clinical utility of hs-cTnT and hs-cTnI measurements depends on the assay generation or on patient characteristics, including age, sex, and indicators of myocardial stress. Although findings in PROSPER and 3 previous studies 8, 25, 26 suggested that the prognostic value of cardiac troponins does not vary according to NT-proBNP levels, analysis of large-scale individual-participant data is required to assess multiplicative interactions adequately.

### Biological mechanisms

The traditional view has been that cardiac troponins are elevated in case of myocardial necrosis only; however, evidence has emerged that other heart diseases such as atrial fibrillation (43) and cardiac structural and functional abnormalities 15, 44 can also lead to modest increases in cardiac troponin concentrations in circulation. Another relevant factor could be subclinical coronary atherosclerosis. Coronary angiography studies have shown close correlations between the severity of coronary atherosclerosis and cardiac troponin concentration (45). It is speculated that clinically silent ruptures of coronary plaques trigger microembolizations and, consequently, the shedding of troponin from cardiomyocytes via membranous blebs (46). A third mechanism could be the link between cardiac troponin concentration and cardiac stress characterized by activation of the adrenergic and renin-angiotensin-aldosterone systems. This relationship has been demonstrated in patients with heart failure (47).

### Clinical relevance

Our data also suggested that cardiac troponin assessment could be a useful adjunct to conventional risk factors in predicting CVD risk. In line with a preferential association of cardiac troponins with fatal CVD, assessment of hs-cTnT in the PROSPER study yielded greater improvements for predicting fatal CVD than overall CVD. For fatal CVD, it yielded improvements in the C-index as well as categorical and continuous NRIs. For the overall CVD outcome, hs-cTnT assessment only improved the continuous NRI.

Another group of biomarkers that has emerged as a potential adjunct to CVD risk prediction are the natriuretic peptides. In the Natriuretic Peptides Studies Collaboration, a large collaborative individual-participant-data meta-analysis, NT-proBNP demonstrated associations with CVD (RR: 1.76; 95% CI: 1.56 to 1.98) and CHD (RR: 1.67; 95% CI: 1.45 to 1.93) that were of similar magnitude to those observed in PROSPER for hs-cTnT (48). The association between hs-cTnT and CVD risk was weakened in PROSPER after adjustment for NT-proBNP but remained significant. It might be that a combination of these biomarkers or a more extensive panel of complementary biomarkers will provide the greatest improvement in prediction. However, this approach might be limited by cost, which requires further study.

Furthermore, the data presented from PROSPER and studies in the current meta-analysis all classified participants on the basis of a single measurement of cardiac troponin. Diurnal variability in cardiac troponin has been observed with moderately higher levels during the morning (49), and short-term variability was present both in those with and without known coronary artery disease (50). This biological variation will need to be explored further to inform selection of appropriate cutoffs for use in clinical practice. Evaluations of the prognostic value of changes in cardiac troponin over time have suggested that the trajectory of cardiac troponin might provide additional prognostic information 12, 51.

Recent data from the WOSCOPS (West of Scotland Coronary Prevention Study) trial showed that pravastatin significantly lowered hs-cTnI in a manner consistent with lower CVD risk (28). This recent paper, along with our summation of available prospective data on troponins and CVD risk, suggests that more research on troponins as markers of clinical utility beyond the diagnosis of an acute coronary syndrome is urgently needed.

### Study limitations

This study provided de novo data from the PROSPER study and was strengthened by concurrent reporting of a comprehensive review of the literature regarding the association between cardiac troponin and incident CVD. Transformation of study-specific HRs to reflect RR between those with cardiac troponin values in the upper third compared with the lower third allowed a common scale to be used when pooling studies and has been used in the assessment of a number of biomarkers (52). It did, however, assume that the relationship between the biomarker of interest and disease risk was linear. In the meta-analysis, there was significant between-study heterogeneity that could have resulted from several differences between the included studies as discussed earlier. Inherent limitations exist in the use of study-level data in this meta-analysis, and the availability of individual-participant data would allow for further standardization of analytical techniques, covariate adjustment, and subgroup analysis.

## Conclusions

Elevated cardiac troponin concentration, well within the normal range, was associated with an increased risk of incident CVD outcomes in the general population. This association was strongest for fatal CVD and persisted after adjustment for conventional CVD risk factors. Further research on cardiac troponin as a useful marker of risk prediction seems warranted.

Perspectives **COMPETENCY IN MEDICAL KNOWLEDGE:** Meta-analysis of published studies suggests that people with elevated circulating troponin concentrations in absence of clinically manifest myocardial injury are at a relatively high risk of developing cardiovascular events.

**TRANSLATIONAL OUTLOOK:** Future research is needed to clarify the predictive value of cardiac troponin measurements in relation to conventional cardiovascular risk factors.
