# Supplementary material for: High-Sensitivity Cardiac Troponin Concentration and Risk of First-Ever Cardiovascular Outcomes in 154,052 Participants
Source: J Am Coll Cardiol. 2017 Aug 1;70(5):558–68. doi: 10.1016/j.jacc.2017.05.062 (PMC5527070; doi:10.1016/j.jacc.2017.05.062)
Supplement: Online Data [file mmc1.pdf]

High-sensitivity cardiac troponin concentration and risk of first-ever cardiovascular outcomes: Literature-based meta-analysis involving 154,052 participants

Supplemental Material

SUPPLEMENTAL METHODS

THE PROSPER STUDY.

The Prospective Study of Pravastatin in the Elderly at Risk (PROSPER) is a randomized, double-blind, placebo-controlled trial designed to investigate the effect of pravastatin in prevention of vascular events in older individuals with pre-existing cardiovascular disease or risk factors thereof (1,2). Between 15 December 1997 and 7 May 1999, a total of 5,804 individuals were screened at the study centres in Scotland, Ireland and the Netherlands. To be included participants were required to have: (i) either pre-existing vascular disease (coronary, cerebral, or peripheral) or raised risk of such disease because of smoking, hypertension or diabetes; (ii) a plasma total cholesterol level of 4.0–9.0 mmol/L; and (iii) a triglyceride concentration of ≤6.0 mmol/L. The list of exclusion criteria of PROSPER are provided in the design paper (2) and included: (i) poor cognitive function (Mini-Mental State Examination score <24 points); (ii) congestive heart failure (defined as New York Heart Association functional class III or IV); (iii) a diagnosis of atrial fibrillation, or (iv) abnormal laboratory findings such as serum creatinine of >200 µmol/L. For the purpose of the present investigation, we excluded participants with a history of myocardial infarction or stroke (n=980), missing information on hs-cTnT concentration (n=25) or on covariates (n=397), leaving 4,402 participants in the analysis (Online Figure 1). hs-cTnT concentration was measured in plasma samples obtained six months after randomisation using a high-sensitivity electrochemiluminiscence immunoassay on a Roche Modular Analytics E170 platform. The occurrence of incident outcomes was adjudicated by the PROSPER Endpoints Committee during the in-trial phase (mean duration: 3.2 years) and ascertained with routine health data thereafter. The combined CVD endpoint was composed of fatal coronary heart disease (CHD), non-fatal myocardial infarction, and fatal plus non-fatal stroke (ischemic, hemorrhagic or

unclassified). When re-infarction or death occurs following a non-fatal myocardial infarction within the same period of hospitalization (up to and including 14 days from the date of admission), the subsequent event were regarded as the same event unless electrocardiographic and/or post-mortem evidence suggests an infarction in a different site. When death occurred following a non-fatal stroke within a period of 28 days from the event, it was regarded as due to a fatal stroke in the absence of other clinical events. Information on fatal outcomes was available in the overall cohort, whereas additional information on non-fatal outcomes was available for the Scottish trial centre (n=1,889). The institutional ethics review boards of all centres approved the protocol and all participants gave written informed consent. The protocol adhered to the principles of the Declaration of Helsinki.

REFERENCES

1. Shepherd J, Blauw GJ, Murphy MB, et al. Pravastatin in elderly individuals at risk of vascular disease (PROSPER): a randomised controlled trial. Lancet 2002;360:1623–30.
2. Shepherd J, Blauw GJ, Murphy MB, et al. The design of a prospective study of Pravastatin in the Elderly at Risk (PROSPER). PROSPER Study Group. PROspective Study of Pravastatin in the Elderly at Risk. Am J Cardiol 1999;84:1192–7.

SUPPLEMENTAL TABLES

Online Table 1 Search strategy for the systematic review of the published literature

PubMed
((("high-sensitive" OR "high sensitivity" OR "highly-sensitive" OR "highly sensitivity" OR "sensitive assay") AND troponin OR "sensitive Troponin") AND ("Cardiovascular Diseases"[Mesh] OR "Cardiovascular Disease" OR "Cardiovascular Death" OR "Vascular Disease" OR "Vascular Death" OR "Ischemic Heart Disease" OR "Myocardial ischaemia" OR "Myocardial ischemia" OR "Acute coronary syndrome" OR "Coronary disease" OR "Coronary heart disease" OR "Coronary artery disease" OR "Myocardial infarction" OR "Heart attack" OR "Cerebrovascular disease" OR "Stroke" OR "Apoplexy" OR "Brain vascular accident" OR "Cerebrovascular accident") AND ((cohort studies[MeSH]) OR (epidemiologic studies[MeSH]) OR (prospective studies[MeSH]) OR (epidemiologic stud*) OR (cohort stud*) OR (population stud*) OR (prospective stud*) OR (observational stud*) OR (longitudinal stud*) OR (odds OR risk OR hazard))) NOT ("Animals"[Mesh] NOT "Humans"[Mesh])
Web of Science
TS=(("high-sensitive" OR "high sensitivity" OR "highly-sensitive" OR "highly sensitivity" OR "sensitive assay") AND troponin OR "sensitive Troponin") AND TS=("Cardiovascular Disease" OR "Cardiovascular Death" OR "Vascular Disease" OR "Vascular Death" OR "Ischemic Heart Disease" OR "Myocardial ischaemia" OR "Myocardial ischemia" OR "Acute coronary syndrome" OR "Coronary disease" OR "Coronary heart disease" OR "Coronary artery disease" OR "Myocardial infarction" OR "Heart attack" OR "Cerebrovascular disease" OR "Stroke" OR "Apoplexy" OR "Brain vascular accident" OR "Cerebrovascular accident") AND TS=((epidemiologic stud*) OR (cohort stud*) OR (population stud*) OR (prospective stud*) OR (observational stud*) OR (longitudinal stud*) OR (odds OR risk OR hazard))
EMBASE
(("high-sensitive" OR "high sensitivity" OR "highly-sensitive" OR "highly sensitivity" OR "sensitive assay") AND troponin OR "sensitive Troponin").af AND ("Cardiovascular Disease" OR "Cardiovascular Death" OR "Vascular Disease" OR "Vascular Death" OR "Ischemic Heart Disease" OR "Myocardial ischaemia" OR "Myocardial ischemia" OR "Acute coronary syndrome" OR "Coronary disease" OR "Coronary heart disease" OR "Coronary artery disease" OR "Myocardial infarction" OR "Heart attack" OR "Cerebrovascular disease" OR "Stroke" OR "Apoplexy" OR "Brain vascular accident" OR "Cerebrovascular accident").af AND ((epidemiologic stud*) OR (cohort stud*) OR (population stud*) OR (prospective stud*) OR (observational stud*) OR (longitudinal stud*) OR (odds OR risk OR hazard)).af

Online Table 2 Baseline characteristics of PROSPER participants

Baseline characteristic	Mean (SD), median [IQR] or n (%)				P value*
	Overall	Bottom hs-cTnT third (<5 ng/L)	Middle hs-cTnT third (5-8 ng/L)	Top hs-cTnT third (>8 ng/L)	
No. of participants	4,402	1,375	1,345	1,682	
Questionnaire-based					
Age, years	75 (3)	74 (3)	75 (3)	76 (3)	<0.001
Male sex, n (%)	1973 (45%)	374 (27%)	610 (45%)	989 (59%)	<0.001
Current smoker, n (%)	1237 (28%)	413 (30%)	383 (28%)	441 (26%)	0.004
Intervention group, n (%)	2187 (50%)	675 (49%)	666 (50%)	846 (50%)	1.000
Physical measurements					
Body mass index, kg/m^2	26.8 (4.2)	26.3 (4.0)	27.0 (4.3)	27.2 (4.3)	<0.001
Systolic blood pressure, mmHg	155 (21)	151 (21)	155 (21)	159 (22)	<0.001
Diastolic blood pressure, mmHg	84 (11)	83 (11)	84 (11)	85 (11)	<0.001
Blood-based biomarkers					
NT-proBNP at 6 months, pg/mL	136 [75, 261]	100 [59, 171]	127 [75, 235]	195 [100, 413]	<0.001
Total cholesterol, mmol/L	5.7 (0.9)	5.9 (0.9)	5.7 (0.9)	5.6 (0.9)	0.102
HDL cholesterol, mmol/L	1.3 (0.4)	1.3 (0.3)	1.3 (0.4)	1.3 (0.4)	0.330
eGFR, mL/min/1.73m^2	58 (14)	59 (13)	58 (14)	56 (14)	<0.001
C-reactive protein, mg/L	3.1 [1.5, 6.0]	2.7 [1.4, 5.5]	3.2 [1.6, 6.2]	3.3 [1.7, 6.6]	<0.001
Baseline disease history					
History of diabetes mellitus, n (%)	467 (11%)	110 (8%)	147 (11%)	210 (12%)	0.019
History of hypertension, n (%)	2874 (65%)	856 (62%)	873 (65%)	1145 (68%)	<0.001

*P values for differences across hs-cTnT thirds were estimated using linear or logistic regression models, as appropriate, adjusted for age, sex, centre, and intervention arm. Abbreviations: eGFR=estimated glomerular filtration rate, HDL=high-density lipoprotein, NT-proBNP=N-terminal pro B-type natriuretic peptide.

Online Table 3 Progressive adjustment the associations of hs-cTnT concentration with cardiovascular outcomes in PROSPER

Outcome	Thirds of hs-cTnT concentration (ng/L)		
	Bottom third (<5 ng/L)	Middle third (5-8 ng/L)	Top third (>8 ng/L)
CVD			
No. of events	141	162	216
Hazard ratio (95% CI)			
Model 1	1 [Reference]	1.32 (1.05, 1.66)	1.60 (1.27, 2.02)
Model 2	1 [Reference]	1.31 (1.04, 1.65)	1.55 (1.23, 1.96)
Model 3	1 [Reference]	1.24 (0.98, 1.57)	1.32 (1.03, 1.68)
CVD death			
No. of events	132	192	370
Hazard ratio (95% CI)			
Model 1	1 [Reference]	1.42 (1.14, 1.78)	2.14 (1.74, 2.65)
Model 2	1 [Reference]	1.43 (1.14, 1.79)	2.16 (1.74, 2.67)
Model 3	1 [Reference]	1.38 (1.10, 1.73)	1.83 (1.47, 2.28)
CHD			
No. of events	105	121	179
Hazard ratio (95% CI)			
Model 1	1 [Reference]	1.30 (1.00, 1.70)	1.79 (1.38, 2.33)
Model 2	1 [Reference]	1.32 (1.01, 1.72)	1.85 (1.42, 2.42)
Model 3	1 [Reference]	1.25 (0.96, 1.64)	1.60 (1.21, 2.11)
Stroke			
No. of events	84	83	102
Hazard ratio (95% CI)			
Model 1	1 [Reference]	1.17 (0.85, 1.59)	1.29 (0.94, 1.77)
Model 2	1 [Reference]	1.14 (0.84, 1.56)	1.21 (0.88, 1.67)
Model 3	1 [Reference]	1.12 (0.82, 1.54)	1.11 (0.80, 1.56)

Model 1 was adjusted for age, sex, and centre, and stratified by treatment arm. Model 2 was further adjusted for smoking status, history of diabetes mellitus, history of hypertension, systolic blood pressure, total cholesterol, high-density lipoprotein cholesterol, and body mass index. Model 3 was further adjusted for C-reactive protein, estimated glomerular filtration rate, and N-terminal pro B-type natriuretic peptide.

Online Table 4 Improvements in CVD prediction by addition of information on hs-cTnT concentration to a model containing conventional risk factors in PROSPER*

Risk prediction metric	CVD		Fatal CVD	
	Estimate (95% CI)	P value	Estimate (95% CI)	P value
C-index				
Conventional risk factors	0.593 (0.574, 0.622)	-	0.600 (0.583, 0.616)	-
Addition of hs-cTnT	0.602 (0.584, 0.622)	-	0.628 (0.613, 0.643)	-
Difference in C-index	0.009 (-0.017, 0.037)	0.51	0.028 (0.007, 0.050)	0.018
Categorical NRI†				
Cases	-0.039 (-0.072, -0.005)	0.012	0.108 (0.063, 0.153)	<0.001
Non-Cases	0.063 (0.039, 0.087)	<0.001	0.015 (-0.004, 0.034)	0.059
Overall	0.024 (-0.017, 0.066)	0.25	0.123 (0.074, 0.153)	<0.001
Continuous NRI				
Cases	0.044 (-0.042, 0.130)	0.31	0.210 (0.138, 0.283)	<0.001
Non-Cases	0.108 (0.055, 0.161)	<0.001	0.146 (0.114, 0.178)	<0.001
Overall	0.152 (0.052, 0.253)	0.003	0.357 (0.277, 0.436)	<0.001

*The conventional risk factors model included information on age, sex, centre, smoking status, history of diabetes mellitus, systolic blood pressure, and levels of total cholesterol and high-density lipoprotein cholesterol, and was stratified by treatment arm. Information on hs-cTnT was entered in categories as specified in Figure 1. †Net reclassification across 10-year risk categories <15%, 15%-<25%, and ≥25%.

Online Table 5 Reclassification of PROSPER participants across categories of predicted 10-year risk of CVD and fatal CVD with addition of information on hs-cTnT to a model containing conventional risk factors

(a) CVD

**Conventional risk factors model**	**Addition of information on hs-cTnT**					
	**No. of cases (%)**			**No. of non-cases (%)**		
**Categories of predicted risk**	<15%	15-<25%	≥25%	<15%	15-<25%	≥25%
<15%	9 (1.7)	**4 (0.8)**	**0**	55 (4.0)	**28 (2.0)**	**0**
15-<25%	**4 (0.8)**	97 (18.7)	**26 (5.0)**	**59 (4.3)**	395 (28.8)	**75 (5.5)**
≥25%	**0**	**46 (8.9)**	333 (64.2)	**0**	**130 (9.5)**	628 (45.8)

(b) Fatal CVD

**Conventional risk factors model**	**Addition of information on hs-cTnT**					
	**No. of cases (%)**			**No. of non-cases (%)**		
**Categories of predicted risk**	<15%	15-<25%	≥25%	<15%	15-<25%	≥25%
<15%	143 (20.6)	**73 (10.5)**	**11 (1.6)**	1526 (41.2)	**303 (8.2)**	**28 (0.8)**
15-<25%	**63 (9.1)**	164 (23.6)	**84 (12.1)**	**512 (13.8)**	624 (16.9)	**275 (7.4)**
≥25%	**3 (0.4)**	**27 (3.9)**	126 (18.2)	**23 (0.6)**	**132 (3.6)**	280 (7.6)

Numbers in **green** indicate reclassification in the desired direction (up for cases, down for non-cases). Numbers in **red** indicate reclassification in the undesired direction (down for cases, up for non-cases). The conventional risk factors model included information on age, sex, centre, smoking status, history of diabetes mellitus, systolic blood pressure, and levels of total cholesterol and high-density lipoprotein cholesterol, and was stratified by treatment arm. Information on hs-cTnT was entered in categories as specified in Figure 1.

SUPPLEMENTAL FIGURES

Online Figure 1 STROBE diagram

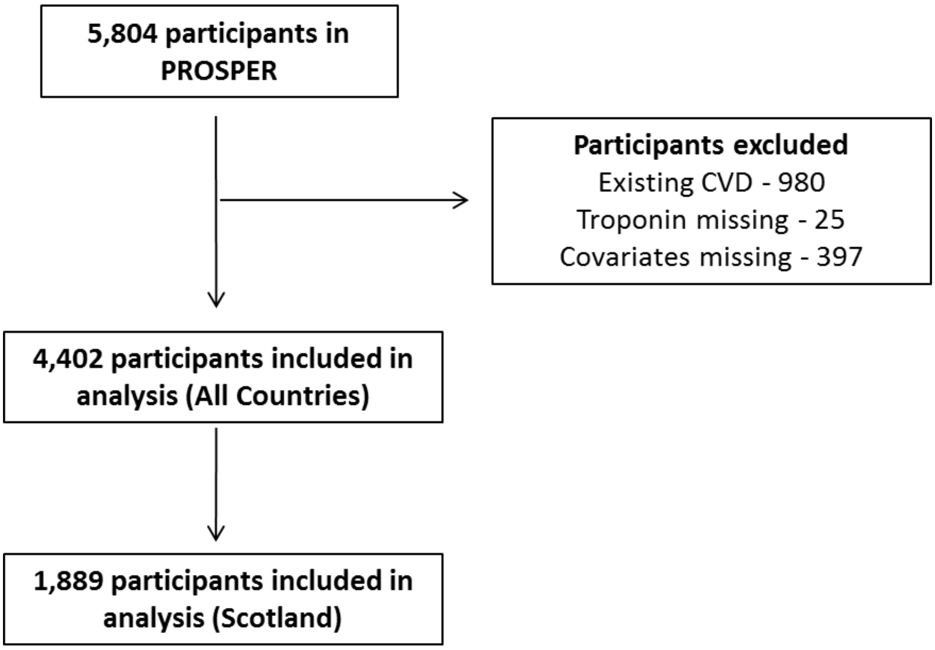

Online Figure 2 Subgroup analysis of associations of hs-cTnT concentration with CVD and fatal CVD in the PROSPER study

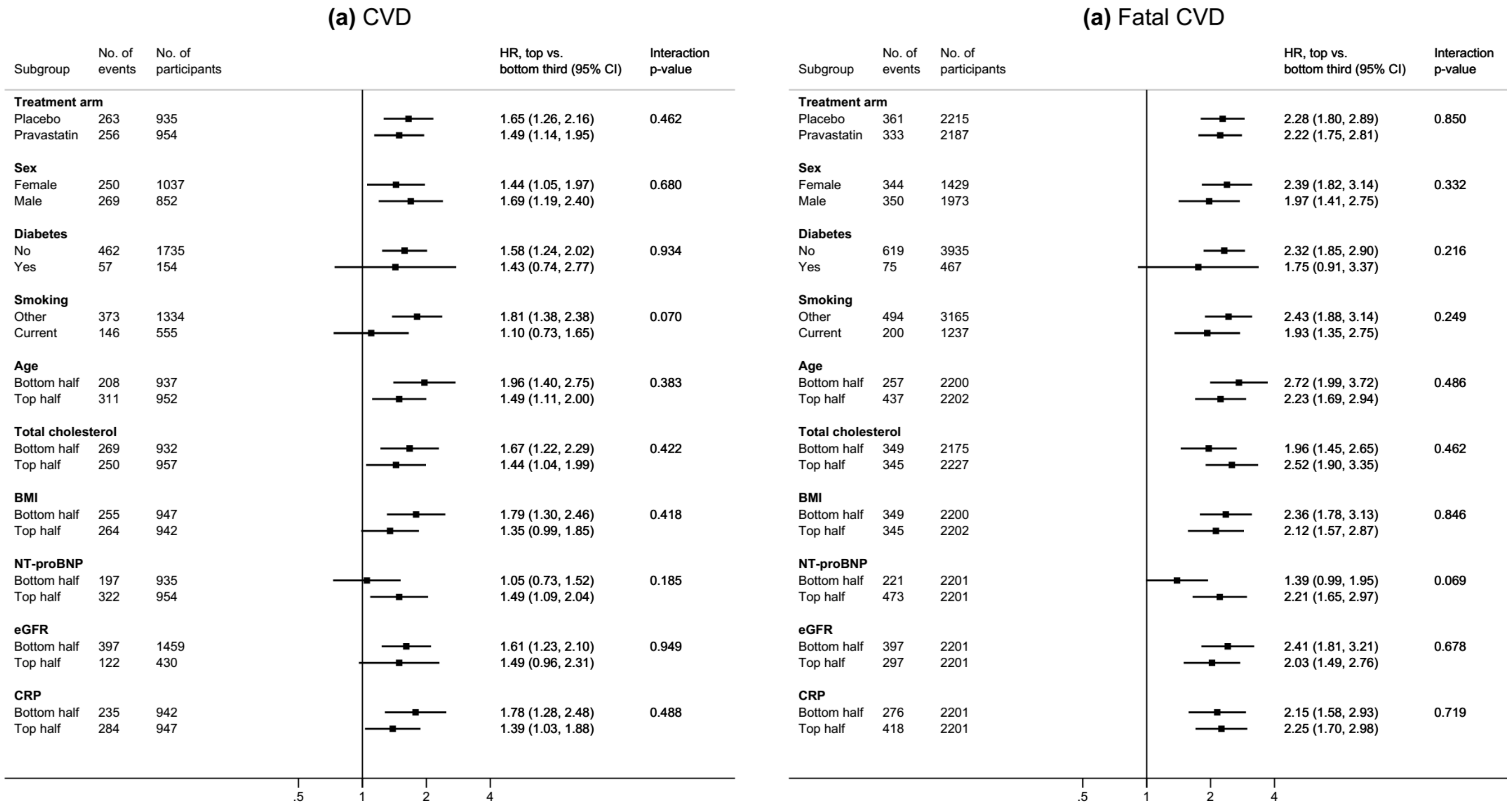

The models were adjusted for age, sex, centre, smoking status, history of diabetes mellitus, history of hypertension, systolic blood pressure, total cholesterol, high-density lipoprotein cholesterol, and body mass index, and stratified by treatment arm. Abbreviations: BMI=body mass index, CRP=C-reactive protein, eGFR=estimated glomerular filtration rate, HR=hazard ratio, NT-proBNP=N-terminal pro B-type natriuretic peptide.

Online Figure 3 Flow diagram of the systematic literature review

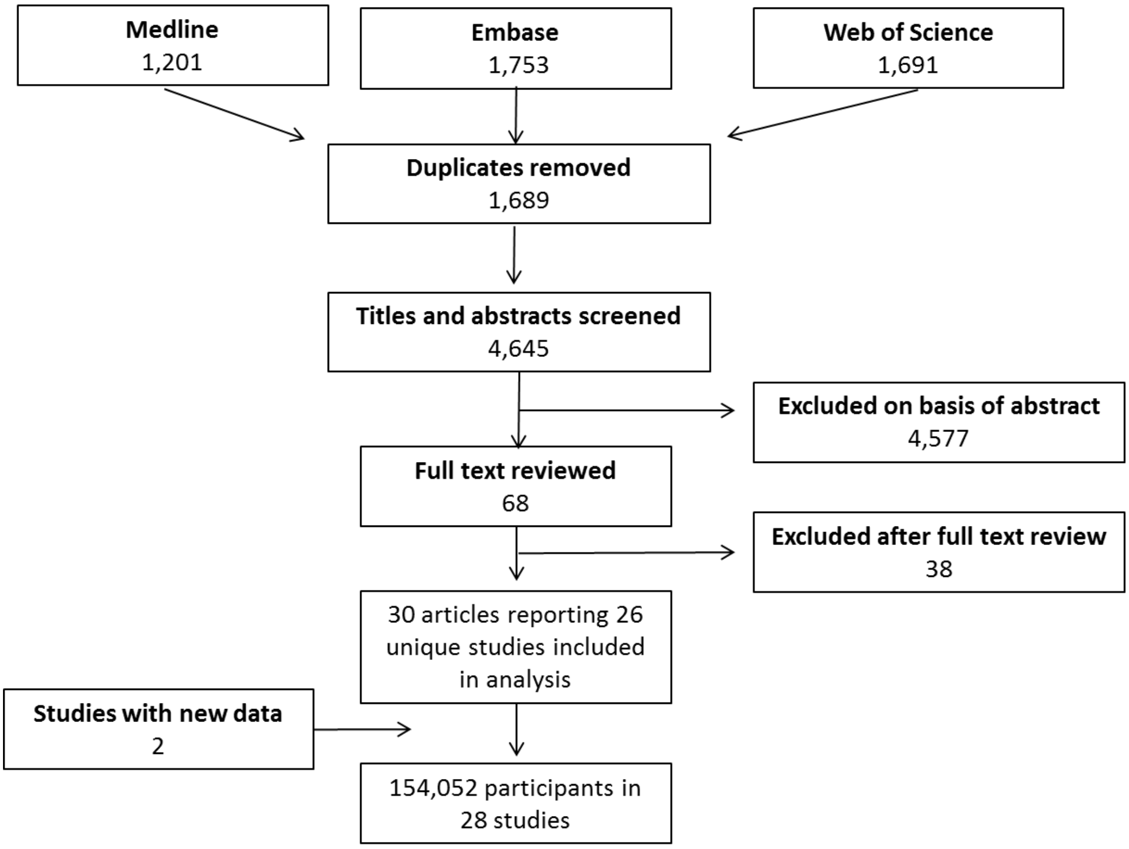

Online Figure 4 Relative risks for cardiovascular disease in individuals in the top compared to the bottom third of cardiac troponin concentration

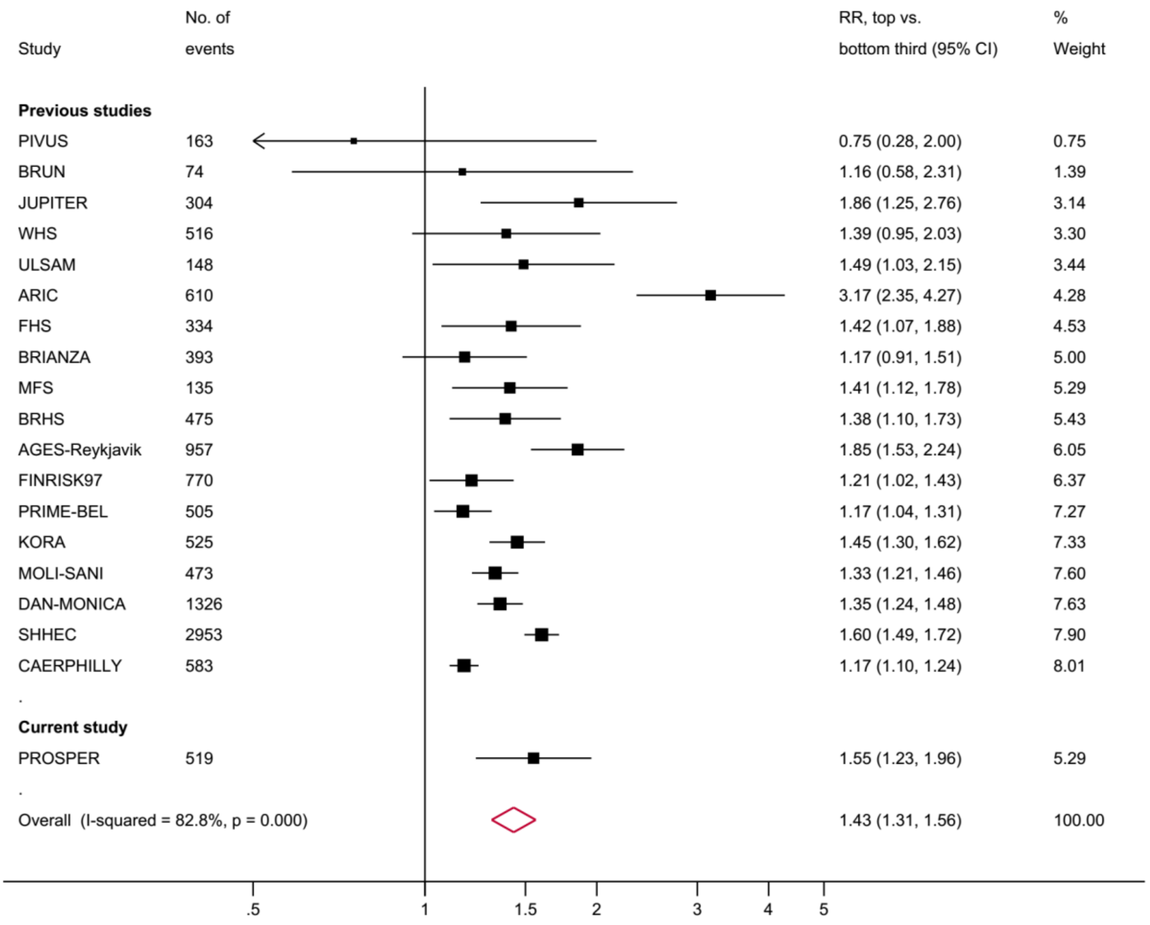

Sizes of data markers indicate the weight of each study in the analysis. For full study names, see footnote to Table 1.

Online Figure 5 Relative risks for coronary heart disease in individuals in the top compared to the bottom third of cardiac troponin concentration

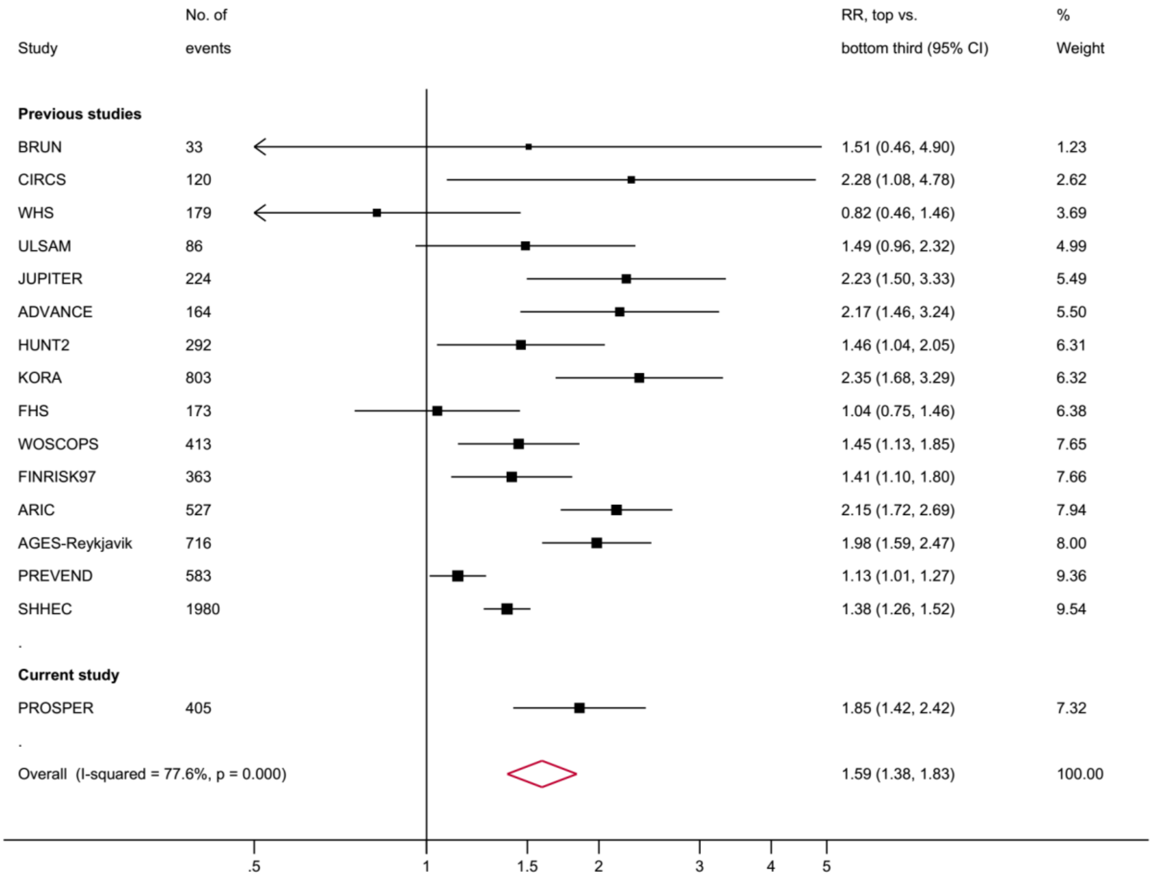

Sizes of data markers indicate the weight of each study in the analysis. For full study names, see footnote to Table 1.

Online Figure 6 Relative risks for stroke in individuals in the top compared to the bottom third of cardiac troponin concentration

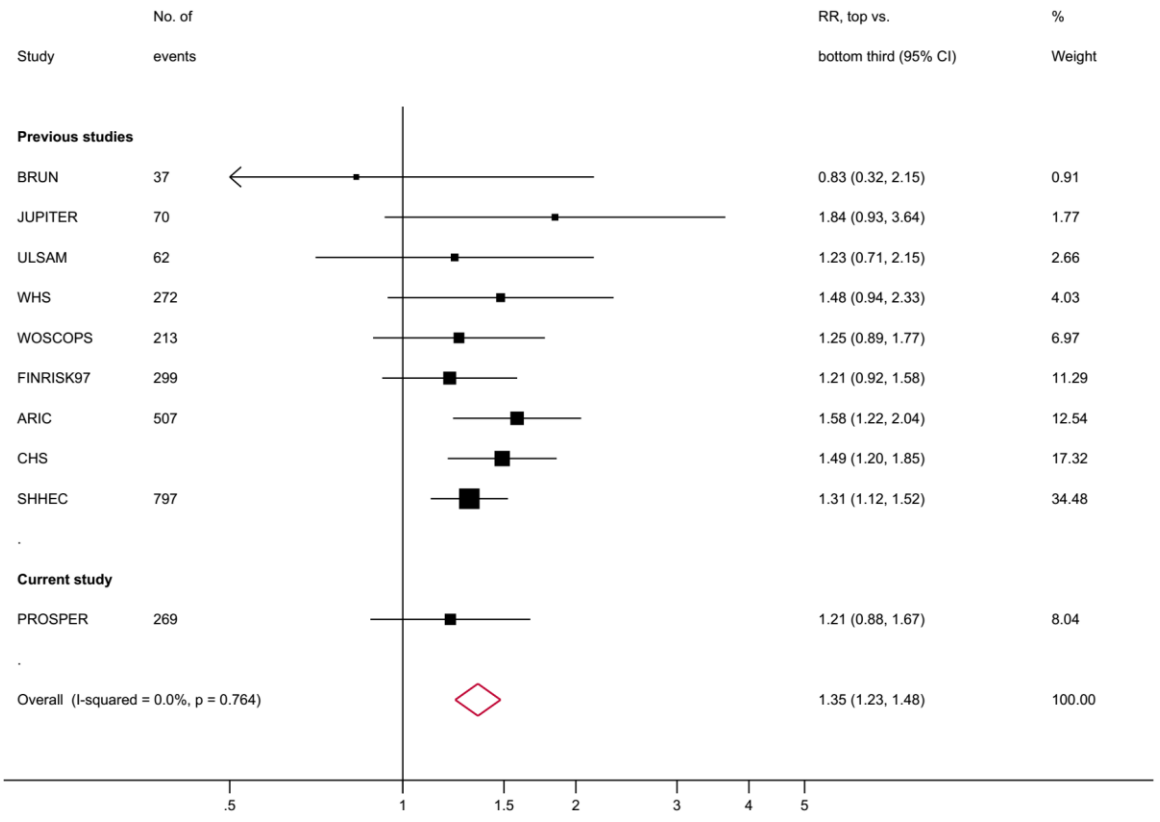

Sizes of data markers indicate the weight of each study in the analysis. For full study names, see footnote to Table 1.

Online Figure 7 Relative risks for fatal cardiovascular disease in individuals in the top compared to the bottom third of cardiac troponin concentration

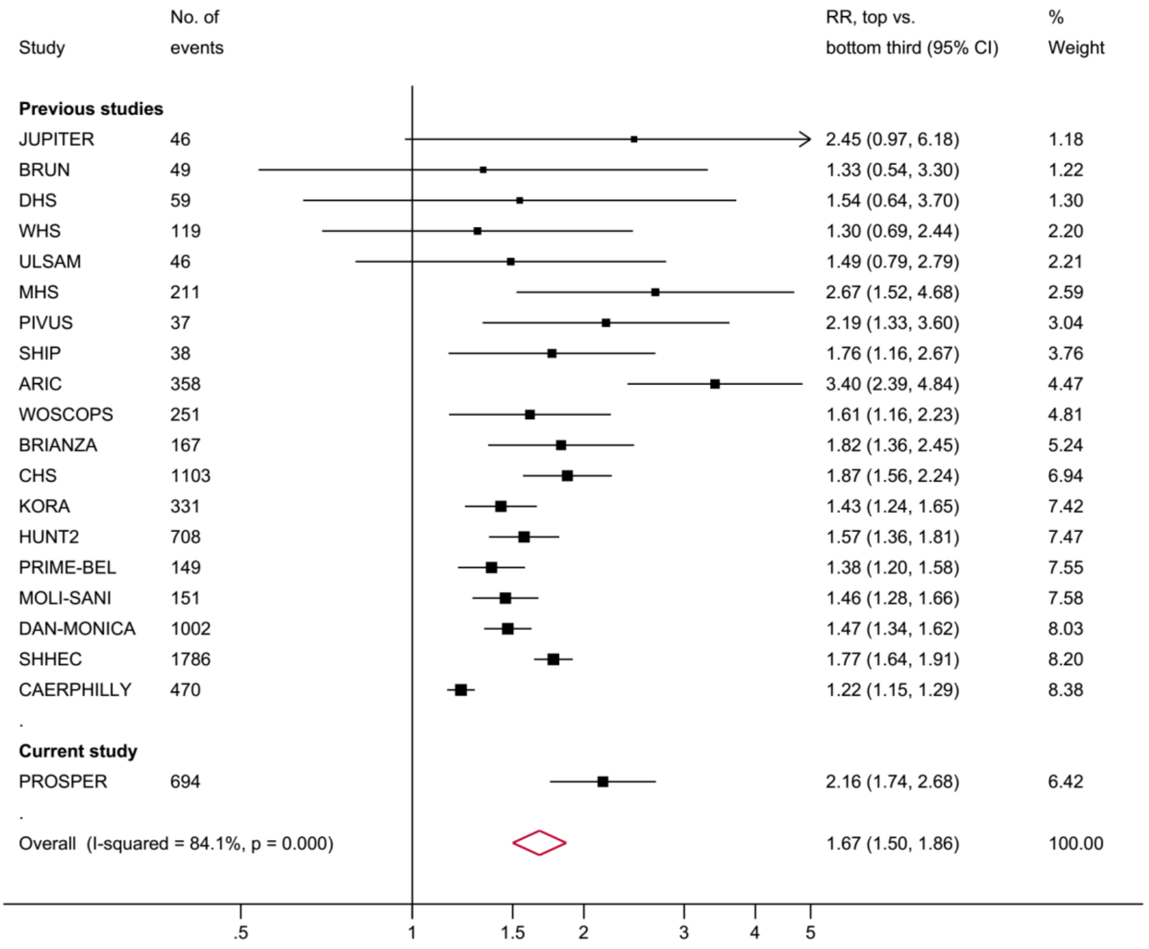

Sizes of data markers indicate the weight of each study in the analysis. For full study names, see footnote to Table 1.

Online Figure 8 Relative risks for cardiovascular outcomes in individuals in the top vs bottom third of cardiac troponin concentration according to categories of study-level characteristics

Subgroup	Category	No. of studies	No. of participants	No. of events	RR, top vs. bottom third (95% CI)	P value*
**CVD**						
Geographical region	Europe	14	86751	9480	1.35 (1.24, 1.48)	0.083
	North America	3	10655	1460	1.85 (1.07, 3.20)	
	Other	2	14845	823	1.62 (1.33, 1.99)	
Study nested in trial	No	16	95889	10424	1.41 (1.28, 1.55)	0.513
	Yes	3	16362	1339	1.57 (1.31, 1.87)	
Troponin type	Troponin I	12	97296	9286	1.36 (1.24, 1.50)	0.171
	Troponin T	7	14955	2477	1.60 (1.27, 2.02)	
Sample type	Serum	10	80900	8559	1.35 (1.22, 1.49)	0.125
	Plasma	9	31351	3204	1.59 (1.31, 1.93)	
Level of adjustment	+	10	83705	8472	1.39 (1.25, 1.56)	0.544
	++	2	7580	1476	1.72 (1.45, 2.04)	
	+++	7	20966	1815	1.42 (1.25, 1.62)	
**Fatal CVD**						
Geographical region	Europe	13	85776	5185	1.52 (1.37, 1.69)	0.010
	North America	5	18960	1850	2.14 (1.52, 3.01)	
	Other	2	17358	740	2.17 (1.76, 2.68)	
Study nested in trial	No	16	100241	6665	1.64 (1.46, 1.84)	0.508
	Yes	4	21853	1110	1.87 (1.49, 2.35)	
Troponin type	Troponin I	13	97993	5347	1.56 (1.40, 1.74)	0.027
	Troponin T	7	24101	2428	1.99 (1.58, 2.50)	
Sample type	Serum	13	89037	6224	1.55 (1.39, 1.73)	0.018
	Plasma	7	33057	1551	2.05 (1.61, 2.61)	
Level of adjustment	+	10	74321	4556	1.53 (1.36, 1.73)	0.107
	++	4	28074	1485	1.90 (1.50, 2.41)	
	+++	6	19699	1734	1.88 (1.36, 2.59)	

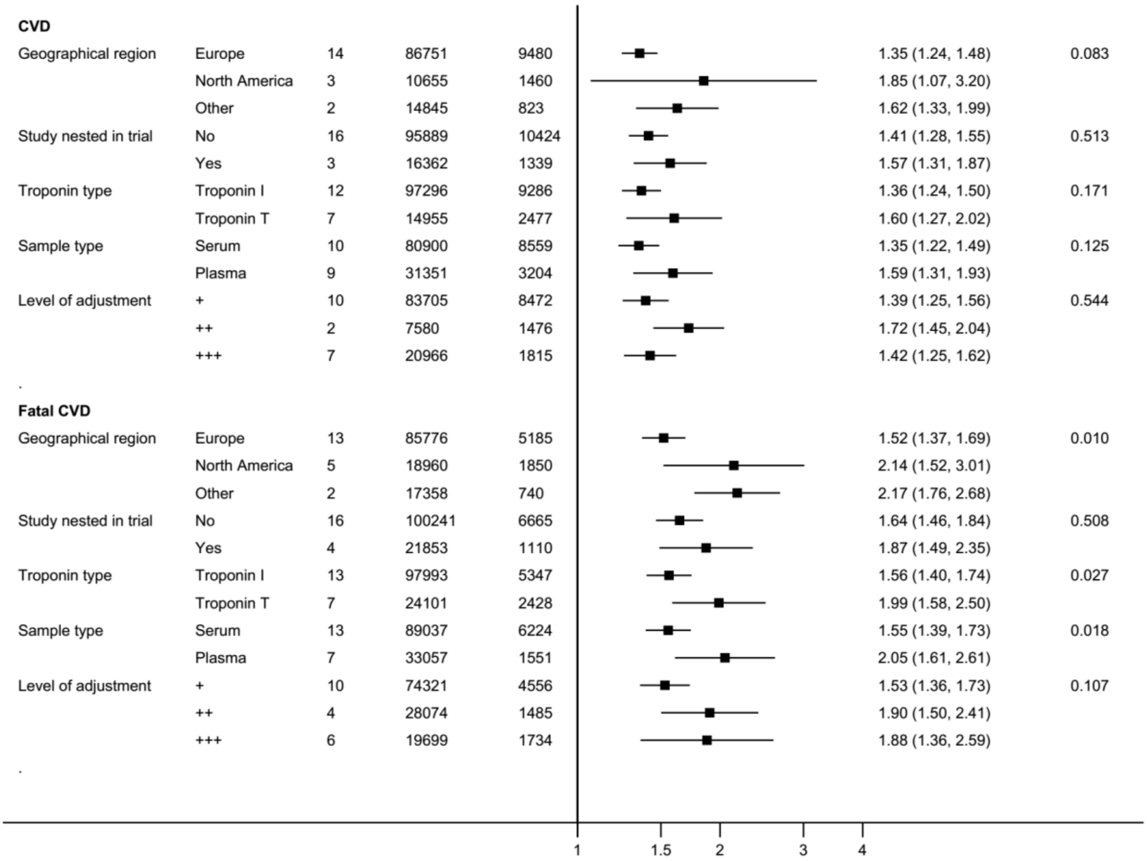

*P values were calculated from meta-regression. Level of adjustment: +, adjusted for age and sex; ++, adjusted for age, sex, and smoking status; +++, adjusted for age, sex, smoking status, and other established CHD risk factors.

Online Figure 9 **Relative risks for cardiovascular outcomes in individuals in the top vs bottom third of cardiac troponin concentration according to continuous study-level characteristics**

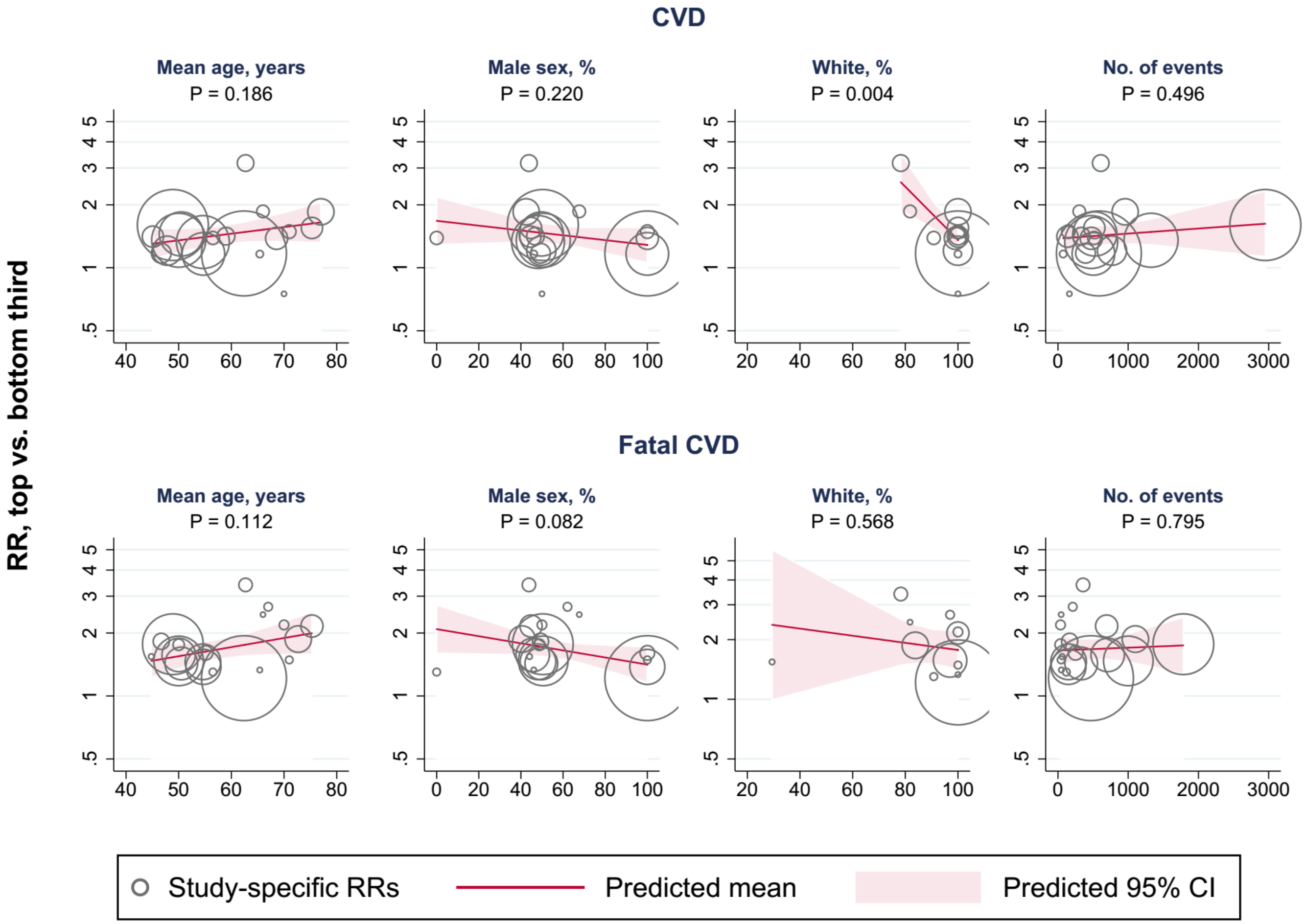

Each circle represents one study; the size of the circle is proportional to the inverse variance of the study-specific relative risk. P values were calculated from meta-regression.

Online Figure 10 Funnel plots showing reported associations of cardiac troponin concentration with risk of cardiovascular outcomes

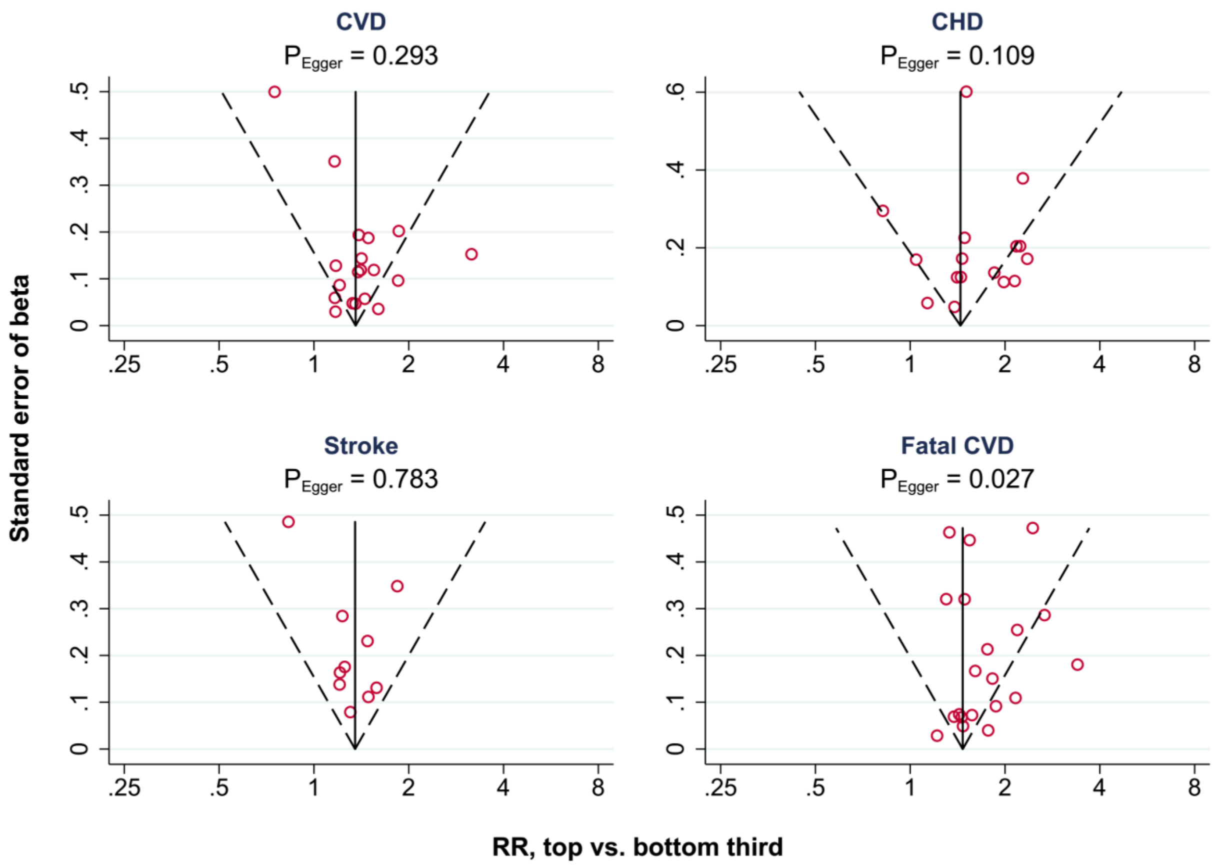

Funnel plots show study-specific relative risks plotted against their standard error. Each circle represents one study. In the absence of publication bias, studies lie within the symmetric funnel, with studies with high precision lying near the average effect and those with low precision spread evenly on both sides of the average effect. Visual deviation can indicate publication bias. P values from the Egger-test test for presence of publication bias. CHD=coronary heart disease, CVD=cardiovascular disease, RR=relative risk.
